# Iron dyshomeostasis links obesity and neurological diseases

**DOI:** 10.4103/NRR.NRR-D-24-01657

**Published:** 2025-04-29

**Authors:** Bandy Chen

**Affiliations:** Department of Medicine, UC San Diego School of Medicine, La Jolla, CA, USA

With the industrialization of agriculture and the advancement of medical care, human life expectancy has increased considerably and continues to rise steadily. This results in novel and unprecedented challenges, namely obesity and neurodegeneration. Understanding the intimate link between these two pathologies will enhance our understanding of brain-body crosstalk to develop effective treatments to halt the rising rates of obesity and neurological disorders. Obesity increases the risk of cognitive decline and neurological disorders; however, it remains unclear the exact mechanisms of obesity-induced neurodegeneration. This perspective proposes iron as a nexus between obesity and neurological disorders through the reshaping of the neuro-glial-vascular landscape.

Obesity is associated with alterations to various cerebral processes including neurovascular coupling, blood–brain barrier (BBB) integrity, cerebrospinal fluid dynamics, and myelination that in conjunction contribute to the increased risk of cognitive decline and neurological disorders. These processes are partly regulated by iron homeostasis, therefore understanding how obesity can shift brain iron homeostasis will elucidate the impact of obesity on the cerebral landscape and promote the repurposing of iron-modulating therapeutics utilized in neurological disorders to treat metabolic diseases.

**Obesity-induced brain iron overload:** Obesity and metabolic diseases such as type 2 diabetes mellitus (T2DM) are associated with systemic iron overload and oxidative stress. Historically, the focus has largely been on peripheral iron metabolism, with brain iron dysregulation unexplored. Recent shifts toward brain iron metabolism have gained momentum due to its implications in neurodegeneration. This is extending towards obesity due to the intimate link between metabolic and neurological diseases. Given the chronic nature of obesity, it is essential to investigate changes in the brain across various phases of nutrient overload. Hypothalamic iron concentration positively correlates with body weight, blood glucose, and HOMA-IR (Zhang et al., 2024). In mice, short-term high-fat diet (HFD) feeding for 2 weeks increases hypothalamic iron concentration, indicating hypothalamic iron overload as an early event and contributes to the development of obesity (Zhang et al., 2024). Iron overload is prevalent in agouti-related peptide (AgRP)-expressing neurons in the arcuate nucleus of the hypothalamus. This leads to elevated oxidative and endoplasmic reticulum stress in AgRP neurons, resulting in central insulin and leptin resistance. Iron depletion in AgRP neurons inhibits their activity and ameliorates diet-induced obesity and associated metabolic dysfunction. Intracerebroventricular administration of the iron chelator deferoxamine ameliorates dietary obesity and its associated metabolic disorders through AgRP neurons. This brings into question whether iron dyshomeostasis is due to adiposity or nutrient composition of HFD. Leptin-deficient *ob/ob* mouse demonstrates elevated hypothalamic iron concentration, hinting that iron overload is associated with obesity rather than the nutrient composition of HFD.

Spanning past the hypothalamus, the hippocampus of *ob/ob* mice exhibits elevated iron overload-related mitochondrial ferritin compared to wild-type mice (Jin et al., 2020). Similarly, leptin receptor-deficient *db/db* mice has increased iron accumulation and altered iron metabolism in the hippocampus (Wang et al., 2024b). In T2DM mice, iron content is significantly elevated in the cortex with increased lipid peroxidation in the hippocampus (Guo et al., 2023; Wang et al., 2024a). Additionally, T2DM rats demonstrate hippocampal iron overload (Liu et al., 2020). Similar effects are seen *in vitro*, where primary neurons and astrocytes exposed to high glucose/palmitic acid develop ferroptosis (Tang et al., 2022; Wang et al., 2024b). Follow-up studies on the role of high glucose on other cell types of the neuro-glial-vascular unit will provide a comprehensive view of the compounding effects of nutrient overload on brain iron and ferroptosis. Interestingly, the impact of HFD on iron content spans across generations. Maternal HFD feeding results in altered iron metabolism in offspring (Graf et al., 2016). Brain hepcidin, ferroportin, and L-ferritin levels are elevated in whole brain homogenates of maternal HFD offspring. Moreover, in the APP/PS1 mouse model of Alzheimer’s disease, HFD feeding increases nonheme iron overload in the hippocampus and cortex (Xiao et al., 2022). Overall, obesity in various models of metabolic and neurological diseases increases brain iron overload and ferroptosis. Further research focusing on the regional and temporal specificity of iron overload throughout obesity is essential for targeted interventions.

**Brain iron overload alters cognitive function:** Iron deposition and myelin loss are seen throughout the aging brain (Jang et al., 2024). Obesity is associated with increased aging and cognitive decline, therefore iron dyshomeostasis in obesity can explain the higher risk for cognitive decline and neurodegeneration. In aged mice, higher levels of ferritin are associated with lower levels of myelin in the cortex and corpus callosum (Jang et al., 2024). Visceral fat removal decreases ferritin levels in activated microglia/macrophages and mature oligodendrocytes, which ameliorates myelin loss. This indicates that systemic inflammation, especially adipokines, contributes to brain iron overload and demyelination via the upregulation of hepcidin. Further investigation focusing on specific adipokines associated with brain iron overload could enable the development of more targeted interventions. Intriguingly, one of the earliest links between obesity and cognitive deficits is demonstrated by the *ob/ob* mouse, where the lack of leptin leads to massive adiposity, neurodevelopment abnormalities, brain-wide myelin loss, and cognitive impairments. The hippocampus of the *ob/ob* mouse exhibits decreased volume and elevated neuroinflammation, BBB impairments, and tau phosphorylation (Jin et al., 2020). A unique marker that is robustly elevated in the serum and hippocampus of the *ob/ob* mouse is lipocalin-2, an adipokine that regulates iron homeostasis. The proposed mechanism is that elevated lipocalin-2 worsens neuroinflammation, iron overload, and oxidative stress, which leads to neurodegeneration in obese conditions. This aligns with studies that demonstrate the ability of lipocalin-2 to inhibit remyelination. Moreover, lipocalin-2 is implicated in various neurological diseases such as Alzheimer’s disease, reinforcing the role of peripheral adiposity on neurological health. The mechanisms of lipocalin-2’s effect linking iron homeostasis and myelination require further elucidation; however, it remains a promising target for obesity-induced neurodegeneration. Additional models of obesity exhibit a comparable pattern of iron-induced neurodegeneration. In maternal HFD offspring, iron overload is associated with myelin loss in the medial and lateral cortices (Graf et al., 2016). These structural changes are linked to neurobehavioral deficits. Similarly, neonatal iron overload induces memory impairments and metabolic dysfunction in rats (do Nascimento et al., 2024). Iron overload leads to increased hippocampal insulin receptor and GLUT1 mRNA expression and decreased AKT phosphorylation, suggesting that iron metabolism interferes with insulin signaling. This implies that brain insulin resistance is a pathway of iron-induced neurodegeneration. HFD potentiates these impairments, indicating the synergistic deleterious effects of early iron overload and HFD exposure on cognitive health (do Nascimento et al., 2024). In summary, both polygenic and monogenic obesity contribute to iron-induced neurodegeneration; however, distinct mechanisms between different models of obesity require clarification.

To understand the mechanisms of iron-induced neurodegeneration, pharmaceutical interventions are utilized to tease out the specific mechanisms involved in brain iron dyshomeostasis. In *db/db* mice, treatment with gemfibrozil, a peroxisome proliferator-activated receptor α agonist, restores hippocampal iron metabolism by inhibiting ferroptosis and lipid peroxidation induced by iron deposition in astrocytes (Wang et al., 2024b). Peroxisome proliferator-activated receptor α activation alleviates brain iron overload through the cystine/glutamate antiporter/glutathione peroxidase 4 regulated ferroptosis pathway. This improves cognitive impairments and reduces neuronal and synaptic loss. In T2DM mice, treatment with erythropoietin, a glycoprotein involved in the production of red blood cells, ameliorates cognitive impairments by decreasing hippocampal iron levels and lipid peroxidation (Guo et al., 2023). Erythropoietin functions as a ferroptosis inhibitor to counteract iron overload, which improves neuronal viability. Likewise, artemisinin, a natural compound derived from the plant Artemisia annua, demonstrates similar benefits for diabetes-associated cognitive dysfunction (Wang et al., 2024a). Artemisinin alleviates neuronal loss and ferroptosis in the hippocampal CA1 region via nuclear factor erythroid-2-related factor 2 (Nrf2) activation. This increases glutathione peroxidase 4/glutathione expression and inhibits lipid peroxidation and iron overload. While myelin content and oligodendrocyte markers were not assessed in the study, Nrf2 is a prominent marker for oligodendrogenesis and myelination. Further studies focusing on the mechanistic link between iron overload, Nrf2, and myelination can elucidate the link between obesity and cognitive decline. Another marker that is downregulated in the hippocampus of T2DM mice and high glucose-cultured primary neurons is caveolin-1, a membrane lipid raft scaffolding protein that is essential for the formation of caveolae (Tang et al., 2022). This aligns with previous works that demonstrate that caveolin-1 is attenuated in the hippocampus of T2DM patients and *db/db* mice. *In vivo*, overexpression of caveolin-1 in T2DM mice attenuates neuronal ferroptosis and ameliorates cognitive impairment by improving mitochondrial and neuronal functions (Tang et al., 2022). The proposed mechanism is through the upregulation of the AMPK/Nrf2/Fpn pathway. Chronic nutrient overload inhibits AMPK phosphorylation and decreases Nrf2 expression in the hippocampus, which can be reversed by caveolin-1 overexpression. This suggests that AMPK activation is a key pathway to inhibit brain iron overload and ferroptosis. Notably, the most prescribed antidiabetic drug metformin enacts its effects through the AMPK pathway and has been shown to be neuroprotective by stimulating remyelination. Hepcidin therapy is a canonical treatment for iron overload diseases, mainly targeting peripheral tissues to exert its effect on systemic iron homeostasis. In T2DM rats, upregulation of brain hepcidin using recombinant adeno-associated virus suppresses hippocampal iron deposition and improves cognitive impairments (Liu et al., 2020). This study demonstrates for the first time that hepcidin therapy can be utilized for brain iron overload to improve neurological deficits in metabolic disorders. This complements preliminary studies that demonstrate hepcidin therapy on iron-mediated neurotoxicity seen in neurodegeneration such as intracerebral hemorrhage.

**Figure 1:**
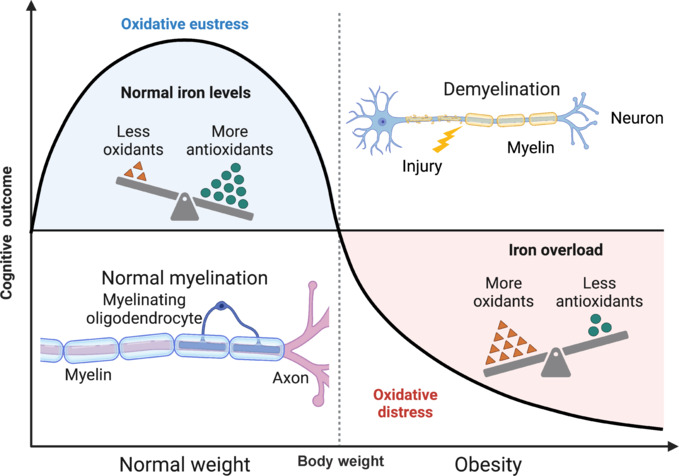
Obesity induces brain iron overload and rewires the myelin landscape. Chronic overnutrition leads to altered iron metabolism and overload, resulting in uncontrolled oxidative stress and neuroinflammation. This leads to the uncoupling of different brain processes such as neurovascular coupling, blood–brain barrier permeability, and cerebrospinal fluid dynamics. The accumulation of cerebral decoupling culminates in demyelination and cognitive decline. Created with BioRender.com.

Obesity and its associated comorbidities increase the risk of vascular diseases and unfavorable functional outcomes and poor recovery after ischemic brain injury. Uncontrolled ferroptosis is predicted to be a primary cause of vasoregression after stroke in diabetic conditions. In T2DM mice, post-stroke treatment with deferoxamine prevents vasoregression and microglia activation and improves aquaporin-4 polarity and BBB permeability in the cortex and striatum (Abdul et al., 2021). For brain microvascular endothelial cells isolated from diabetic mice, deferoxamine prevents iron-mediated decrease in cell viability and lowers the ferroptosis markers IREB2 and ACSL4. The ability of iron chelation to affect different processes of the brain indicates the importance of iron in mediating neuro-glial-vascular unit function. Lastly, HFD APP/PS1 mice demonstrate elevated cognitive deficits, neuroinflammation, and Alzheimer’s disease markers such as amyloid-β (Xiao et al., 2022). Iron chelation with M30 reverses these impairments without affecting hematological parameters in whole blood. While these studies demonstrate the potential of reversing iron overload for the treatment of obesity-induced neurodegeneration (**Figure 1**), it is crucial to assess other parameters in addition to behavioral and cognitive tests such as myelination, BBB integrity, cerebrospinal fluid dynamics, and neuronal activity. All these different processes of the brain work in conjunction to maintain cognitive function, therefore understanding the specific brain iron pathways involved in the regulation of each process in health and disease will provide a reductionist approach to understanding obesity-induced neurodegeneration.

**Conclusions:** Due to the multifactorial causation and the systemic effect of obesity on the brain and body, it has been a challenge to pinpoint the mechanisms of obesity-induced neurodegeneration. Iron overload caused by obesity is a promising target for intervention due to the intimate link between iron and neurological health. The complex interactions of iron with the neuro-glial-vascular unit have yet to be fully understood. Additional research on this topic will be valuable for advancing the understanding and treatment of metabolic and neurological disorders. It is important to pinpoint the transition from health to disease in brain iron metabolism throughout obesity. This will allow the optimization of therapeutic interventions for the prevention and treatment of neurometabolic diseases.
